# Dataset on the environmental and social footprint of the University of the Basque Country UPV/EHU

**DOI:** 10.1016/j.dib.2022.107847

**Published:** 2022-01-20

**Authors:** Gorka Bueno, Maite de Blas, Estibaliz Pérez-Iribarren, Iñaki Zuazo, Eduardo Torre-Pascual, Artitzar Erauskin, Iker Etxano, Unai Tamayo, María García, Ortzi Akizu-Gardoki, Iñigo León, Cristina Marieta, Estibaliz Saez de Cámara, Gaizka Zulueta, Isaac Barrio

**Affiliations:** University of the Basque Country UPV/EHU

**Keywords:** Organisation Environmental Footprint (OEF), Organisational life cycle assessment (O-LCA), Social organisational life cycle assessment (SO-LCA), Higher education institution, Life cycle assessment (LCA)

## Abstract

The organisational life cycle assessment (O-LCA) and the social organisational life cycle assessment (SO-LCA) of the University of the Basque Country UPV/EHU were conducted. The data presented in this paper support the calculation of the environmental and social footprint of the University of the Basque Country UPV/EHU for year 2016 [1], and may be used as a reference for future calculations of the environmental and social footprint of higher education institutions and other organisations.

This dataset provides detailed information on the UPV/EHU and the boundaries considered; on the compilation and quantification of the life cycle inventory (LCI) –which included a transport survey conducted in summer 2018–; and on the modelling process followed for the calculation of the environmental and social footprints, based on the ecoinvent 3.3 database [2] and PSILCA-based Soca v1 add-on [3, 4], and carried out with the openLCA free software [5]. The dataset also includes the life cycle impact assessment (LCIA) results provided by the CML (baseline, 2015) [6] and ReCiPe (endpoint (H), 2008) [7] LCIA methods and post-processed social impacts provided by the Social Impacts Weighting Method [3], disaggregated by subprocesses and impact locations. Data is provided for the reference year (2016), and some aggregated data is also provided for alternative scenarios that were explored in order to check pathways to reduce social and environmental impacts of the academic activity of the UPV/EHU [1].

## Specifications Table

**Table:** 

Subject	Environmental Science
Specific subject area	Organisational & social life cycle assessment
Type of data	Text, tables, figures
How data were acquired	LCI data were collected from primary sources by direct measurement in the UPV/EHU, from invoices issued by service providers, from a survey (provided as a supplementary file in the repository) on transport needs conducted in summer 2018 among staff and students of the UPV/EHU, and from interviews with service providers. Data was also provided by secondary sources, primarily ecoinvent 3.3 database [2] and PSILCA-based Soca v1 add-on [3,4]; values for some social indicators were obtained from public institutions in the Basque Country: UPV/EHU [8,^9^,13], URA [10], Eustat [11], Numbeo [12].
Data format	Raw, analysed and preprocessed data
Parameters for data collection	Primary data were collected for the base year 2016 (calendar year: January-December) when possible, although for some flows it was necessary to take the academic year as a unit (September 2016 - August 2017), or later years. Buildings considered for the data collection where used by 96.8% of total users of the UPV/EHU, excluding Medicine Teaching Units in the Bizkaia and Gipuzkoa Campuses, the Faculty of Engineering (Navigation and Naval Machines) located in Portugalete (Bizkaia), and other entities isolated from other buildings that have less than 25 users. LCI data were gathered for consumption (electricity, fuels, main materials and products), the generation of waste (urban waste, hazardous waste, electrical and electronic equipment waste, wastewater) and transport services demanded by users, which included daily commuting, change of residence at weekends and punctual work displacements for staff.
Description of data collection	Primary data were collated via face-to-face, telephone and email communications with service providers and UPV/EHU administrators, and via a survey conducted in 2018 in the UPV/EHU (followed by data treatment). In some cases data was obtained by projection from other faculties or complemented with educated guesses. Secondary data were collated through access to the commercial ecoinvent 3.3 database [2] and PSILCA-based Soca v1 add-on [3,4] using openLCA [5], and through access to reports from public institutions and sources [8], [9], [10], [11], [12], [13].
Data source location	Institution: University of the Basque Country UPV/EHU Region: Autonomous Community of the Basque Country Country: Spain GPS coordinates: Leioa campus [43.3312169,-2.9719147] Faculty of Engineering (Bilbao) [43.2624212,-2.9501195] Faculty of Business and Economics (Sarriko, Bilbao) [43.273274,-2.9591532] Faculty of Business and Economics (Elkano, Bilbao) [43.2602222,-2.9337763] Vitoria-Gasteiz campus [42.8357225,-2.6844644] Donostia-San Sebastián campus [43.3072221,-2.0099339] Faculty of Engineering (Eibar) [43.179452,-2.4911498]
Data accessibility	Data are available within the article and detailed supplementary information is also provided through a repository. Repository name: Mendeley Data Data identification number: doi:10.17632/y4zz26pdzk.3 Direct URL to data: http://dx.doi.org/10.17632/y4zz26pdzk.3
Related research article	G. Bueno, M. de Blas, E. Pérez-Iribarren, I. Zuazo, E. Torre-Pascual, A. Erauskin, I. Etxano, U. Tamayo, M. García, O. Akizu-Gardoki, I. León, C. Marieta, G. Zulueta, I. Barrio, The environmental and social footprint of the university of the Basque Country UPV/EHU. J Clean Prod 315:128019. https://doi.org/10.1016/J.JCLEPRO.2021.128019

## Value of the Data

• These data provide information about the organisational environmental and social footprint of a higher education institution.
• These data are useful for any LCA practitioner interested in calculating the environmental and social footprint of an organisation, and in particular of a university.
• These data provide a valuable reference for the combined calculation of the environmental and social footprint of a higher education institution and other organisations.
• These data are valuable in gathering information on the transport needs of organisations and on the transport habits within them.
• The data related to the calculation of social impacts provide valuable information on the interpretation of results provided by the PSILCA-based Soca add-on.

## 1 Data Description

This dataset provides all primary and secondary data used to build the organisational life cycle assessment (O-LCA) and the social organisational life cycle assessment (SO-LCA) of the University of the Basque Country UPV/EHU for year 2016. The LCI data presented support the original research carried out, firstly, to calculate the UPV/EHU's environmental and social footprint for year 2016, and secondly, to explore pathways to reduce harmful environmental impacts derived from its academic activities [1].

Table 1 provides background data related to the UPV/EHU in year 2016 and to the alternative scenarios that were analysed to explore possible impact reduction pathways. This background data includes the characterisation of the electricity mix supplied to the UPV/EHU and other parameters that are modified in the alternative scenarios, such as the lifespan of computer infrastructure, the consumption of electricity from renewable sources, the provision of thermal energy by heat pumps, and the shift of part of users’ road transport from private cars to buses. Table 2 provides data related to other four more specific transport scenarios also explored in the original research [14].

**Table 1:** Background data related to the UPV/EHU in year 2016 and to the alternative scenarios analysed to explore possible impact reduction pathways.

	UPV/EHU year 2016	Scenario A Computer lifespan extended	Scenario B Electricity 100% renewable	Scenario C Transport shifted from car to bus/coach	Scenario D heat production by heat pumps	Scenario E heat production by heat pumps powered with renewable electricity
Electricity mix	Electricity suppplied by EDP Comercializadora SAU (36.4% from fossil fuels and 20.3% of nuclear origin) [15] (see Table A.3)	As in 2016	Electricity suppplied is 100% of renewable origin, following the high voltage mix provided by ecoinvent v3.3 for Spain in 2016 (see Table A.3).	As in 2016		Electricity suppplied is 100% of renewable origin, following the high voltage mix provided by ecoinvent v3.3 for Spain in 2016 (see Table A.3).

Lifespan of computer infrastructure	Average lifespan of desktops and laptops is seven years; average lifespan of displays is 14 years.	Average lifespans of desktops, laptops and displays are extended by two years.	As in 2016			

Shift of transport from private car to bus/coach	Distribution of total transport for students and staff in different modes according to results from the survey conducted in summer 2018 among users (23.2% of total transport needs, measured in terms of passenger-kilometres).	As in 2016		A change in transport habits transfers half of the transport made by car in year 2016 to bus and coach.	As in 2016	

Provision of thermal energy to buildings	Heating is provided in almost all buildings by natural gas boilers, and in some centres by gas-oil boilers.	As in 2016			Natural gas and gas-oil boilers used for thermal energy production are replaced by air-source heat pumps that use R134a as the working fluid and have a seasonal coefficient of operation of 4.	

Occupancy rates for transport modes	Occupancy rates in private cars are those provided by the transport survey: 2.05 passengers per car for students and 1.85 passengers per car for staff. Occupancy rates in transport modes different from the private car are equal to those assumed by ecoinvent v3.3 for the corresponding transport modes.

**Table 2:** Data related to specific transport scenarios analysed in order to improve sustainable mobility in the UPV/EHU.

	UPV/EHU year 2016	Scenario A Occupancy rate doubling	Scenario B Suppression of private vehicles	Scenario C Reduced presence on campus, 4-day week	Scenario D Change to a residence closer to the campus
Average occupancy rate in private cars used by staff	2.05 passengers per car	4.1 passengers per car	As in 2016		

Average occupancy rate in private cars used by staff	1.85 passengers per car	3.7 passengers per car

Shift of transport from private car to bus/coach	Distribution of total transport for students and staff in different modes according to results from the survey conducted in summer 2018 among users (23.2% of total transport needs, measured in terms of passenger-kilometres).	As in 2016	All transport carried out in private vehicles is transferred to other modes of public land transport, following the modal distribution that characterises each user group in each centre of the UPV/EHU considered.	As in 2016	

Presence on campus	The weekly attendance of users to the campus is that obtained from the survey conducted in summer 2018 among users (86% of students and 73% of staff attend five days per week; Figure B.7.2 in the repository).	As in 2016		The number of working-days on campus will be reduced by one day a week, which is replaced by teleworking and online teaching-learning.	As in 2016

Location of residence	Distance of the residence to the campus is that obtained from the survey conducted in summer 2018 among users (60% of total daily commuting correspond to distances shorter than 15 km, and 75% to distances shorter than 40 km. The average displacement to attend the university on a usual day is 27 km for students and 34 km for staff).	As in 2016			Users who travel daily from a residence located at 50 km far or more from the campus move to a residence closer to the campus during the week. This assumes that the new mix of annual transport per moved user is equivalent to the average of the rest of the users living closer to the campus.

### 1.1 Primary data

The UPV/EHU is the public university of the Autonomous Community of the Basque Country, located on the northern coast of Spain. Its 30 faculties and schools are distributed among three campuses, one for each of the three provinces of the Basque Country: the Campus of Araba (located in Vitoria-Gasteiz), the Campus of Bizkaia (Leioa, Bilbao, and Portugalete), and the Campus of Gipuzkoa (Donostia-San Sebastián and Eibar). In the 2016-17 academic year, 68 Bachelor's degrees, 111 Official postgraduate Master's courses, 65 PhD programmes, and 34 own qualifications were offered [8].

In section A_LCI_and_modelling_data of the repository, Table A.1 from file “1_Faculties-centres-buildings and users.docx” provides information on the UPV/EHU centres included in the inventory and the number of users in each of them in year 2016 [8]. Table A.2 from file “2_Inventory of flows.docx” gathers the flows of energy and material consumption, waste generation and transportation needs that were inventoried for the year of reference at the UPV/EHU, and the strategy followed to collect the data.

In section B_LCI_data_for_transport of the repository, Table B.1 from file “1_Transport survey.docx” shows an English version of the transport survey that was conducted by the Directorate of Sustainability of the UPV/EHU among the academic community in summer 2018. The survey was offered in two languages, Basque and Spanish, and therefore there are two sets of responses, which were processed in parallel. The excel files named “2_Responses to mobility survey in Spanish.xlsx” and “3_Responses to mobility survey in Basque.xlsx” provide the raw data used from the survey. File named “4_Data pre-processing and data clearing.docx” details the Python data pre-processing and data clearing carried out with the survey data. The subsequent results are shown in a single spreadsheet in the excel file named (“5_Pre-processed and cleared primary data from survey.xlsx”). These results constitute the primary data from which all transport calculations are made.

### 1.2 Data on the modelling

In section A_LCI_and_modelling_data of the repository, Tables A.3 from file “3_processes from ecoinvent v3.3.docx” and Table A.4 from file “4_transport modes from ecoinvent v3.3.docx” gather the sub-processes considered in the model regarding waste management, materials and products, energy production and supply, water supply (Table A.3), and transport (Table A.4), and their corresponding processes in ecoinvent 3.3 [2], with the necessary adjustments. Table A.5 from file “5_car mix specifications.docx” provides the percentages considered for the passenger car mix used in the modelling of transport needs [14], and Table A.6 from file “6_impact factors for transport modes.docx” gathers the impact factors, extracted from the ecoinvent 3.3 database, that are used for the calculation of environmental impacts of transport at the UPV/EHU [22]. Tables A.7.1-2 from file “7_parameters of the modelling.docx” provide the list of initial and auxiliary parameters considered in the model implemented with the openLCA software [5]. The excel file named “8_LCI of campuses and isolated faculties.xlsx” provides de LCI data for each of three campuses (Leioa, Gasteiz and Donostia) and four isolated centres (Faculties of Engineering in Bilbao and Eibar, and Faculties of Business and Economics in Sarriko and Elkano, in Bilbao) that were considered in the assessment. GPS coordinates for each campus or centre are also provided.

### 1.3 Secondary data on transport

This subsection provides valuable data about the transport needs of users at the UPV/EHU. In section B_LCI_data_for_transport of the repository, Tables B.6.1-4 from file “6_Transport inventory per campus.docx” provide the distribution of annual kilometres travelled by users according to transport modes, types of displacement and user groups, for each of the campuses (Table B.6.1 for the Leioa campus, Table B.6.2 for the centres in Bilbao, Table B.6.3 for the Vitoria-Gasteiz campus, and Table B.6.4 for the Donostia-San Sebastián campus). From file “7_Data on daily commuting.docx”, Table B.7.1 provides the annual kilometres travelled for daily commuting by an average traveller in each campus; Figure B.7.1 shows the distribution of distances travelled for residence change, by students and staff; Figure B.7.2 shows the percentage of weekly attendance by number of days, for students and staff; and Table B.7.2 provides, in relation to daily commuting journeys, average distances travelled (mean and standard deviation) by users for each transport mode considered, and the distributions within each gender group and within each age group.

### 1.4 LCIA data

LCIA results gathered in the dataset include the midpoint impact categories of the CML (baseline, 2015) method [6] and the endpoint categories of the ReCiPe (endpoint (H), 2008) method [7]. Table 3 provides information on the LCIA methods and impact categories considered. The eight categories indicated with an asterisk (*) have been subjected to a more detailed analysis, breaking them down according to the nature of the processes involved, as well as the geographical location of the impacts.

**Table 3:** LCIA methods and impact categories considered in the assessment. Modified from Table 5 in the related research article [1].

Method	Impact category	Unit
CML (Baseline, 2015) [6]	Terrestrial ecotoxicity – TETP inf	kg 1.4-dichlorobenzene eq
	Ozone layer depletion – ODP steady state	kg CFC-11 eq
	* Climate change – GWP100 (IPCC, 2007)	t CO_2_ eq
	* Photochemical oxidation – high NO_x_	kg ethylene eq
	Acidification potential – average Europe	kg SO_2_ eq
	Eutrophication – generic	kg PO_4_^3−^ eq
	Marine aquatic ecotoxicity – MAETP inf	kg 1.4-dichlorobenzene eq
	Depletion of abiotic resources – fossil fuels	GJ
	* Human toxicity – HTP inf	t 1.4-dichlorobenzene eq
	* Depletion of abiotic resources – elements, ultimate reserves	kg antimony eq
	* Freshwater aquatic ecotoxicity – FAETP inf	t 1.4-dichlorobenzene eq

ReCiPe Endpoint (H), 2008 [7]	* Human Health – total	DALY (Disability Adjusted Life Year)
	* Resources – total	$
	* Ecosystems – total	species•yr

In section C_LCIA_data of the repository, Tables C.1 from file “1_environmental impacts of UPV-EHU in 2016 and alternative scenarios.docx” gathers the impacts for each of the environmental categories of CML and ReCiPe LCIA methods for the reference scenario (year 2016) and for the five alternative scenarios that have been explored (see Table 2): Scenario A, in which the lifespan of computer equipment is extended by two years; Scenario B, where electricity supplied to the UPV/EHU is of 100% renewable origin; Scenario C, in which a change in transport habits transfers half of the transport made by private car to bus and coach; Scenario D, where natural gas and gas-oil boilers used for thermal energy production are replaced by air-source heat pumps, which use R134a as the working fluid and have a seasonal coefficient of operation of four; and Scenario E, in which Scenario B and Scenario D are combined (heat pumps are powered with electricity of renewable origin).

Tables C.2.1-16 from file “2_breakdown of environmental impacts in selected categories.docx” provide, on one hand, the contribution of transportation, energy and material consumption and waste treatment to total impacts, and on the other hand, the location of those impacts –divided into three regions: the Basque Country, outside of the Basque Country, and not defined–, for the UPV/EHU as a whole, and for each of the campuses and isolated faculties.

In relation to the contribution of transportation, Table C.3 from file “3_transport impacts per capita.docx” gathers total impacts derived from the satisfaction of transport needs at the UPV/EHU, distributed by campuses and in per capita terms. A more detailed analysis of impacts derived from transport is shown in Tables C.4.1-15 from file “4_environmental impacts of transport.docx”, where the impact distribution in each category is disaggregated according to transport modes, types of displacement and user groups (students and staff). Figure C.4.1 in the same file shows graphically the distribution of environmental impacts by transport modes, and Table C.4.16 presents the total impacts derived from transport for each of the specific scenarios related to transport (see Table 2) that are analysed in order to explore viable pathways to reduce total transport impact (Scenario A, doubling of occupancy rates in private vehicles; Scenario B, suppression of private vehicles; Scenario C, reduced presence on campus, 4-day week; Scenario D, change to a residence closer to the campus).

In relation to the assessment of social impacts, Table C.5 in file “5_characterization of social impact indicators.docx” provides the characterisation of the social impact indicators needed for the post-processing of the results provided by the PSILCA-based Soca v1 add-on module [3, 4] and the openLCA software. Table C.5 is divided into four stakeholders (Workers, Local Community, Society and Value chain actors); for each social impact indicator, this table presents a short definition, the unit of measurement provided by Soca, and the characterisation of the impact factors in each risk level (range and central value assumed in the post-processing of our modelling) [16]. In file “6_social impacts.docx”, Table C.6 presents the results for the post-processing of the social impacts covered by the Soca module. Figures C.6.1 shows the relative contribution of activity hours, weighted by risk level, in relation to transportation, energy and material consumption, waste treatment and labour activity in the UPV/EHU, and in relation to the location of the impact, for some selected social impact categories. Figure C.6.2 shows the relative contribution of activity hours, weighted by risk level, in relation to the location of the impact, for the same selected social impact categories. The excel file “7_post-processing of social impacts.xlsx” provides data on the post-processing of social impacts: the social impacts provided by the Social Impacts Weighting method (in the “Social_impact_results” tab); the inventory results for social impacts by risk level (“Impacts_by_risk_level” tab); the post-processing of the results, following [11] (“Post-processing_of_results” tab); and the calculation of the relative contribution of activity hours, weighted by risk level, according to subprocesses and locations, for selected impact categories (in the rest of the tabs, one for each social impact indicator analysed).

## 2 Experimental Design, Materials and Methods

The purpose of the work that gathered this dataset was to carry out the O-LCA and the SO-LCA of the University of the Basque Country UPV/EHU, and to explore pathways in order to reduce harmful environmental impacts derived from its academic activities. This work has followed the recommendations provided by the European Commission [17] and the UNEP/SETAC initiative [18], which align with ISO 14040, ISO 14044 and with ISO/TS 14072. The goal of the life cycle inventory in this work was set to obtain and present the inventory data linked to the academic activities of the reporting organisation, the UPV/EHU, in the reference year (2016). The scope of the study covered all academic activities taking place within buildings gathered in Table A.1 from the repository; these buildings, which are considered the reporting unit, were used in 2016 by 45,306 users, accounting for 96.8% of total users of the UPV/EHU. The reporting flow was the academic activity performed in these buildings of the UPV/EHU in year 2016. Primary data presented in the previous section was processed in a modelling that is described in the remainder of this section.

In order to collect LCI data, both consumption (electricity, fuels, main materials and products) and the generation of waste (urban waste, hazardous waste, electrical and electronic equipment waste, wastewater) were taken into account. Some flows (electricity, gas, water, hazardous waste) were systematically quantified by direct measurement in the UPV/EHU. The rest were estimated from the data provided by agents responsible for maintenance, canteen and cleaning services or by the administrators of the UPV/EHU facilities, collated via face-to-face, telephone and email communications; some data were supported by invoices issued by service providers. The year 2016 was considered as the base year (calendar year: January-December), although for some flows it was necessary to take the academic year as a unit (September 2016 - August 2017), or later years. In some cases data was obtained by projection from other faculties, or complemented with educated guesses. LCI data quality, consistency and completeness are recorded for documentary purposes using an ecoinvent “pedigree matrix” [19]. This information is gathered in column E (Data quality entry) in each tab in the excel file named “8_LCI of campuses and isolated faculties.xlsx”, from the repository. Table A.2 from the repository gathers the flows of energy and material consumption, waste generation and transportation needs that were inventoried for the year of reference at the UPV/EHU, and the strategy followed to collect the data. Transportation data linked to the academic activities at the UPV/EHU were obtained from a survey conducted by the Directorate of Sustainability of the UPV/EHU among the academic community in summer 2018, as detailed data for year 2016 was not available [20]. This survey, gathered in Table B.1 from the repository, was answered by 2,966 students (error margin of 1.7%) and 603 staff members (error margin of 3.8%). The data from the mobility survey were subjected to a Python data pre-processing and data clearing, as explained in file “4_Data pre-processing and data clearing.docx” from B_LCI_data_for_transport of the repository. The survey characterised the daily commuting of users to and from University centres (80% of total transport needs, measured in passenger-kilometre), changes of residence at weekends (11% of total) and punctual work displacements.

Secondary LCI data were collated through access to the commercial ecoinvent 3.3 database [2] using openLCA [5]. Secondary data for the SO-LCA was provided by the PSILCA-based Soca v1 add-on [3, 4] to the ecoinvent database. The social data linked to labour activity in the UPV/EHU was obtained from the data available for the Basque Country –with information provided by public institutions in the Basque Country and public sources: UPV/EHU [8, 9, 13], URA [10], Eustat [11], Numbeo [12]–, and otherwise assuming the data available for Spain.

The modelling of the processes that give rise to each of the inventoried flows were selected from among the processes available in the ecoinvent v3.3 database –using the Cut-Off approach, according to which the producer of a recyclable material does not receive any credit [21]. Tables A.3-5 from the repository gather the sub-processes considered in the model and their corresponding process in ecoinvent together with the appropriate adjustments to adapt them to the context of the UPV/EHU (electricity mix, efficiencies of combustion equipment, location). Some of these sub-processes are parameterised with the items gathered in tables A.7.1-2. The modification of some of these specific parameters allows for the exploration of other possible scenarios in order to reduce harmful environmental impacts derived from the academic activities of the UPV/EHU. These scenarios were specified in Tables 1 and 2.

The post-processing of social impacts was performed as follows. The results of the LCIA of social impacts are provided by the Soca add-on module to the ecoinvent database in the form of ``risk hours'', according to different levels of risk (from non-existent to very high; see Table C.5 in the repository). Total social impacts are provided by Soca in terms of equivalent medium risk hours, for which it considers in its calculations arbitrary equivalences across the different risk levels, normally applying a factor of ten (x10) between adjacent risk levels. These results, provided by the Social Impacts Weighting Method, are gathered in the repository in the “Social_impact_results” tab of the excel file “7_post-processing of social impacts.xlsx”. Our work followed an alternative strategy that avoided the assumption of arbitrary equivalencies between adjacent risk levels. Our analysis recalculated the impact in each category for each economic sector and region involved in the life-cycle, as the central impact value considered by PSILCA for each level of risk [4] (these values are gathered in Table C.5 from the repository). This calculation allows to calculate the final aggregate social impacts in such a way that, although approximate, is also more accurate than when assuming arbitrary risk level equivalences. This post-processing of risk hours provided by Soca and openLCA was performed for each of the 53 social impact indicators defined by the Social Impact Weighting Method, as shown in the “Post-processing_of_results” tab in the previously mentioned excel file. These social impact indicators provide information of two different kinds. While some indicators provide information on the direct impact related to the socio-economic activity involved in the life-cycle of the UPV/EHU –e.g. the impact categories related to accidents at work, or the economic costs– other social indicators, which we label as indirect social impact indicators, provide information on the socio-economic context of the life-cycle under study –such as illiteracy, social spending on health, child labour or a gender wage gap–. The divergence of this characterisation from that corresponding to the local context of the UPV/EHU –the Basque Country– can be interpreted as an indicator of indirect social impacts –the social footprint– of the life-cycle of the UPV/EHU.

The post-processing of LCIA data also included a dissagregated analysis of environmental and social impacts according to the relative contribution of transport, energy consumption, consumption of material products and the generation of waste and its treatment, and according to the location of the impacts (inside the Basque Country, outside the Basque Country, and in not defined locations). The selected impact indicators for which this disaggregated analysis was performed are those marked with an asterisk in Table 3 (eight environmental categories) and in Table C.6 from the repository (ten social impact indicators, and costs indicator).

## Ethics Statement

This paper does not involve studies with animals and humans. The work of this paper meets the ethical requirements for publication in Data in Brief (https://www.elsevier.com/authors/journal-authors/policies-and-ethics). The authors declare that the transport survey carried out for this work did not require evaluation or approval from the Ethics Committee of the University of the Basque Country (UPV/EHU), since the data collection was done anonymously and online. Implicit informed consent was obtained from the participants because they agreed to answer the questionnaire on a voluntary basis.

## CRediT Author Statement

**Gorka Bueno:** Conceptualization, Methodology, Writing – original draft, Investigation; **Maite de Blas:** Methodology, Writing – review & editing, Investigation; **Estibaliz Pérez-Iribarren:** Methodology, Writing – review & editing, Investigation; **Iñaki Zuazo:** Methodology, Writing – review & editing, Investigation; **Eduardo Torre-Pascual:** Methodology, Writing – review & editing, Investigation; **Artitzar Erauskin:** Methodology, Writing – review & editing, Investigation; **Iker Etxano:** Writing – review & editing, Investigation; **Unai Tamayo:** Writing – review & editing, Investigation; **Maria García:** Investigation; **Ortzi Akizu-Gardoki:** Investigation; **Iñigo León:** Investigation; **Cristina Marieta:** Investigation; **Estibaliz Saez de Cámara:** Investigation; **Gaizka Zulueta:** Investigation; **Isaac Barrio:** Investigation.

## Declaration of Competing Interest

The authors declare that they have no known competing financial interests or personal relationships which have, or could be perceived to have, influenced the work reported in this article.
